# Supplementary material for: A workflow to visualize vertebrate eyes in 3D
Source: PLoS One. 2023 Aug 22;18(8):e0290420. doi: 10.1371/journal.pone.0290420 (PMC10443858; doi:10.1371/journal.pone.0290420)
Supplement: Attachment — Submitted filename: Response to Reviewers.doc [file pone.0290420.s001.doc]

Aachen, XX.XX.2023

PLOS ONE

The Editor

PONE-D-23-15660

Response to Reviewers

Dear Editor. Dear Reviewers.

Thank you very much for your careful evaluation of our initial manuscript on “A workflow to visualize vertebrate eyes in 3D”. We are thankful for your criticism and suggestions and will explain in the following every issue that you raised and each change we made in the manuscript.

**In general.** We changed the format of page 1 according to the requirements of the journal.

In the whole manuscript we changed the format of the headlines and the references to figures and tables according to the requirements of the journal. We corrected all spelling errors, we found. We corrected the naming of the files providing the figures for this manuscript and deleted the indications for placing the figures in the manuscript. We also changed the captions of each figure according to the requirements of the journal.

**Specific changes.** page 2, l 22. We added the word “pseudorealistic” to make clear that the obtained model is based on calculations and not on real stack data.

In the abstract we changed the text in the Conclusion section l. 26-28 according to the recommendation of Reviewer 2 and we believe that the new text is more precise and reflect the findings in this work better.

Page 5, section Histology. The paragraph “The corneal diameter … OCT measurements.” was deleted to reduce redundancy because the biometric measurement methodology is explained on page 11 “Biometry…”. In this section we deleted the sentence “Where means are provided …” because in the revised manuscript we only cite literature where the variation of the measurements were given.

In the caption of Fig.5 (page 12) we deleted the term “small animal eyes” and inserted the species names “mouse”, ”rat”, and “rabbit”.

In table 4 we now included the original data of our measurements and reformatted the table accordingly and following the recommendation of Reviewer 2. We updated the data based on literature data presenting the variation of the measurements and updated Fig.5 accordingly.

On page 13, l.4-5 we add the sentence “A pointcloud as shown in Fig.8 …” to make clear how the figure was generated.

The “Discussion” section was re-written, starting with a paragraph summarizing similar approaches not only in ophthalmology but also in other fields of medicine (page 15, l.16 ff) as requested by Reviewer 1.

On page 18, l. 19 ff we re-wrote the section on study limitations. Here we also commented on the problem of variations in biometric data of individual eyes of experimental animals p18, l 22 ff as it was correctly requested by Reviewer 1. We fully agree with this comment.

On page 19 we added a section “Conclusions” as recommended by Reviewer 2 to briefly summarize our findings and to describe what we found in our work and for what it can be used.

As recommended by the Editor we changed the “Acknowledgment” section deleting all information on the funding source because it is described elsewhere.

The list of References was updated according to changes in the manuscript.

**Specific questions.** Reviewer 1 commented “As the authors also mentioned in the Discussion, more detailed observation of internal structures is possible when ultrasound is used alone/combined. Is there a reason why it was not used in this experiment?”

Authors answer: We stated in the discussion text p16, l13-19 that conventional ultrasound has a lower resolution than light microscopy, only ultrasound biomicroscopy using 50 Mhz scanners may provide images with a similar resolution. However, the depth of these systems is not big enough to use this technique for the posterior parts of the eye like the retina. Furthermore the available systems are handheld, so that a precise scanning to obtain equidistant slices is not possible.

Reviewer 2 wrote: “1. Authors mention that original historical data have problems to create 3D model, and then use a single sagittal section to deal with problems. Please consider to show the comparison of 3D-models based on these two strategies in order to indicate their superiority or inferiority.”

Authors answer: We tried to show this already in the original manuscript with the comparison of fig 6 and 7. Here we compared the two possibilities of obtaining the 3D model in a mouse eye and even after aligning, registration and other image processing steps the 3D model showed considerable artifacts. Using the stack data from larger eyes as from rabbits or rats yielded a 3D model with many more artifacts and distortion.

Reviewer 2 wrote: “Maybe, your goal is to establish standard 3D-eye model for each experimental animal. Because your strategy is based on histological data, your model cannot be available for planning surgical procedures for individual subjects (modeling could not be prior to surgery). How about the variations in ocular dimensions of your subjects and deliverable 3D models? Are your models (Figure 7-11) available as standard models?
Authors answer: You are absolutely right and we included this problem in the discussion section, where we listed the limitations of the study (s.a). An impression of the variation can be found in Fig.5. The standard deviation of the biometric data is approx. 5 % for each parameter. The variation can be due to individual differences of the eye anatomy but also to a variable response to the histological processing.

Again, we like to thank the Editor and the Reviewers for carefully evaluating this manuscript, for suggesting important issues and for giving us the chance to submit this revision. We hope that our response and the corresponding changes in the manuscript are helpful in improving the quality of this paper.

Sincerely, on behalf of the authors.

Peter Walter
